# COVID-19 Vaccines and Autoimmune Hematologic Disorders

**DOI:** 10.3390/vaccines10060961

**Published:** 2022-06-16

**Authors:** María Eva Mingot-Castellano, Nora Butta, Mariana Canaro, María del Carmen Gómez del Castillo Solano, Blanca Sánchez-González, Reyes Jiménez-Bárcenas, Cristina Pascual-Izquierdo, Gonzalo Caballero-Navarro, Laura Entrena Ureña, Tomás José González-López, on behalf of the GEPTI

**Affiliations:** 1 Hematology Department, Hospital Universitario Virgen del Rocío, Instituto de Biomedicina de Sevilla (IBIS/CSIC), 41013 Sevilla, Spain; 2 Hospital Universitario La Paz-IdiPAZ, 28046 Madrid, Spain; nvbutta@gmail.com; 3 Hematology Department, Hospital Universitario Son Espases, 07210 Palma, Spain; mcanaro@gmail.com; 4 Hematology Department, Hospital General de la Coruña, 15006 A Coruña, Spain; ma.del.carmen.gomez.del.castillo.solano@sergas.es; 5 Hematology Department, Hospital del Mar, 08003 Barcelona, Spain; bsanchezgonzalez@parcdesalutmar.cat; 6 Hematology Department, Hospital Serranía de Ronda, 29400 Ronsa, Spain; rejimbar@hotmail.com; 7 Department of Hematology, Gregorio Marañón General University Hospital (HGUGM) Madrid, Instituto de Investigación Gregorio Marañón, 28009 Madrid, Spain; crisizquierdo3@yahoo.es; 8 Hematology Department, Hospital Universitario Miguel Servet, 50009 Zaragoza, Spain; gcaballeron@salud.aragon.es; 9 Hematology Department, Hospital Universitario Virgen de las Nieves, 18014 Granada, Spain; laura_eu@hotmail.com; 10 Hematology Department, Hospital Universitario de Burgos, 09006 Burgos, Spain; tjgonzalez@saludcastillayleon.es; 11 Grupo Español de PTI, 28040 Madrid, Spain; memingot@gmail.com

**Keywords:** COVID-19, vaccines, ITP, VITT, TTP, AIHA and Evans syndrome, antiphospholipid syndrome, catastrophic antiphospholipid syndrome

## Abstract

Worldwide vaccination against SARS-CoV-2 has allowed the detection of hematologic autoimmune complications. Adverse events (AEs) of this nature had been previously observed in association with other vaccines. The underlying mechanisms are not totally understood, although mimicry between viral and self-antigens plays a relevant role. It is important to remark that, although the incidence of these AEs is extremely low, their evolution may lead to life-threatening scenarios if treatment is not readily initiated. Hematologic autoimmune AEs have been associated with both mRNA and adenoviral vector-based SARS-CoV-2 vaccines. The main reported entities are secondary immune thrombocytopenia, immune thrombotic thrombocytopenic purpura, autoimmune hemolytic anemia, Evans syndrome, and a newly described disorder, so-called vaccine-induced immune thrombotic thrombocytopenia (VITT). The hallmark of VITT is the presence of anti-platelet factor 4 autoantibodies able to trigger platelet activation. Patients with VITT present with thrombocytopenia and may develop thrombosis in unusual locations such as cerebral beds. The management of hematologic autoimmune AEs does not differ significantly from that of these disorders in a non-vaccine context, thus addressing autoantibody production and bleeding/thromboembolic risk. This means that clinicians must be aware of their distinctive signs in order to diagnose them and initiate treatment as soon as possible.

## 1. Introduction

The exceptional scenario conditioned by the sudden outbreak of SARS-CoV-2 infection all over the world prompted the search for vaccines that are efficient and easy to manufacture at huge scale. Extensive research led to the production of more than 20 compounds that successfully passed phase III trials and, thus, were deemed to be safe. Nevertheless, the extended use in the real-world of drugs that have successfully passed the test of clinical trials often involves the emergence of adverse events (AEs). Although these are infrequent and will not tip the balance towards product withdrawal, they can be serious and their occurrence has to be envisaged. Indeed, this phenomenon occurred when COVID-19 vaccines started being administered throughout the world. Statements documenting AEs started being reported. Although regular updates repeatedly demonstrate that the majority of these episodes are mild and transient, serious AEs (SAEs) are occasionally described that, albeit rarely, may be fatal. These SAEs are particularly of an autoimmune nature. This is not unexpected, since humoral immune response to vaccination involves the production of antibodies against foreign antigens that, occasionally, could mimic self-ones. Structures often affected by autoimmune disorders include platelets, RBCs and blood vessels. Thus, it is not surprising that hematologic autoimmune disorders are associated with SARS-CoV-2 vaccines, either the mRNA or adenoviral vector-based ones.

When a hematologic autoimmune disorder develops following vaccination, a prompt and accurate diagnosis is essential to initiate the appropriate treatment and avoid bleeding or thromboembolic complications that may sometimes be life-threatening. The main autoimmune disorders of a hematologic nature that have been linked to SARS-CoV-2 vaccination are addressed herein. Commonly used therapies and outcomes are revised. Where suggested, potentially involved mechanisms linking vaccines and autoimmune pathology are described. Finally, guidelines to follow in order to promptly recognize and manage these complications are provided.

## 2. Methods

In order to select the eligible studies whose results would be considered a comprehensive update about the AEs of hematologic autoimmune nature reported in the context of COVID-19 infection, the authors searched the PubMed registry and the regular updates of the European Database of Suspected Adverse Drug Reaction (EudraVigilance), United Kingdom Medicines & Healthcare Products Released Regulatory Agency (MHRA), Health-Infobase Government of Canada, and the Vaccine Adverse Events Reporting System (VAERS). The search criteria consisted of considering all reports regarding phase III clinical trials and the real-world use of vaccines, stating that any hematologic AE had occurred. The latest search date included was 9 May 2022. Although some reports were limited to enumerating AEs, the scarcity of available literature prompted us to consider all statements addressing the occurrence of hematologic AEs, even if the information was incomplete.

The chosen keywords or word combinations to track the information of interest were COVID-19, vaccines, ITP, VITT, TTP, AIHA, Antiphospholipid and Evans syndrome, autoimmune disorder, thrombocytopenia, and anemia.

## 3. Commercially Available COVID-19 Vaccines

More than 100 vaccines have been developed against SARS-CoV-2, 26 of which have been evaluated in phase III clinical trials according to the World Health Organization (WHO) [1]. These can be stratified into up to five different categories according to how they generate immunity. The classically used methods based on whole inactivated or live attenuated virus, although effective, have always raised concerns. Incomplete viral inactivation may not be discarded, and every batch needs to be checked [2]. The development of antibody disease enhancement (ADE) syndrome due to non-neutralizing antibodies may be a risk which, in the COVID-19 scenario, can increase lung pathology [3]. Furthermore, there was a need for quickly available, massively produced vaccines. Nucleic acid-based vaccines constituted an attractive alternative option. They are easier to manufacture, their lipid nanoparticle-based platform is robust and efficient, and can be rapidly produced at industrial scale. For these reasons, they are being widely used, at least in Western countries, in spite of the challenge raised by the poor stability of mRNA-based products that limits storage periods and may compromise in vivo activity [4].

The aforementioned categories are inactivated-virus vaccines, mRNA vaccines, DNA vaccines, viral vector vaccines and protein-based vaccines. Inactivated-virus vaccines consist of whole viruses that cannot infect cells and replicate. Viruses can be inactivated by formalin, heat irradiation or chemical treatment. Moderate immunogenicity can be a drawback, and adjuvants and/or booster doses are required. mRNA vaccines [5] contain the mRNA of the antigen of interest, which enters cells and is translated into the corresponding protein to induce an immune response. DNA vaccines use a plasmid DNA vector to induce response and they are easier to store and the route of administration may not be exclusively intramuscular, but also intradermal or oral. Viral vector vaccines are based on viral delivery systems that contain a nucleic acid which encodes the antigen of interest. Adenoviruses, which are often employed, have also been used as non-enveloped double stranded DNA (dsDNA) for gene therapy purposes, because the encoded gene can be delivered into human cells without integrating in the human genome [6]. Finally, protein-based vaccines are made of fragments of proteins or polysaccharides. The source can be either the target virus, from which the protein is purified, a recombinant protein, virus-infected cells, or virus-like particles. Low immunogenicity can be one disadvantage, and adding an adjuvant might be needed [7]. Supplementary Table S1 shows which category each one of the 26 COVID-19 vaccines that have successfully passed phase III trials belongs to. Table 1 shows the main features of the most widely used ones.

Key concepts

- There are currently many different ways of producing vaccines.
- Obstacles or disadvantages may be storage, stability, or low immunogenicity.

## 4. International Reports of Adverse Events Associated with COVID-19 Vaccines

COVID-19 vaccination programmes are being rolled out globally. Nevertheless, the need for ready availability prompted the approval of vaccines whose studies regarding side effects and efficacy involved shorter than desirable follow-up periods. As time goes on, cases of moderate to severe adverse events (AEs) associated to SARS-CoV-2 vaccines in the real world are being documented. This information is being analyzed and updated regularly, and reports are being made available via databases and agencies such as the European Database of Suspected Adverse Drug Reaction (EudraVigilance), the United Kingdom Medicines & Healthcare Products Released Regulatory Agency (MHRA), the Health-Infobase Government of Canada, or the Vaccine Adverse Events Reporting System (VAERS). AEs are always classified according to the Common Toxicity Criteria (CTC) categories [8,9,10,11]. Between December 2020 and March 2022, 1,114,386,127 doses of four different COVID-19 vaccines, two of them mRNA-based and the other two using adenovirus as vectors, were administered in the European Union, United Kingdom and Canada.

Table 2 depicts the amount of doses administered up to March 2022, including frequency and types of common AEs and serious AEs (SAEs) corresponding to each type of vaccine, according to the information released by the aforementioned databases and articles concerning clinical trials [8,9,10,11,12,13,14,15]. Global numbers of common AEs are not particularly remarkable [12,13,14,15]. On the other hand, the incidence of SAEs is not different than that expected in a worldwide campaign [8,9,10,11]. The SAEs reported so far include, among others, autoimmune disorders, predominantly of hematologic origin, which are addressed in detail below, venous or arterial thrombotic events, paroxysmal arrhythmias or anaphylactic reactions [8,9,10,11]. Reports regarding other vaccines less used in Western countries, such as Gam-COVID-Vac, Ad5-nCoV, NVX-CoV2373, EpiVacCorona, BBIBP- CorV, WIBP- CorV or CoronaVac, identify flu-like illness, injection site pain, headache, asthenia, pain, soreness, fatigue, headache, malaise, myalgia or fever among mild to moderate AEs. SAEs have not been reported except in the case of Gam-COVID-Vac, whose use has been associated with renal colic, deep venous thrombosis (DVT) or extremity abscess development [16].

Key concepts

- AEs are generally mild or moderate.
- Albeit very infrequently, there can be SAEs that need to be paid attention to.

### 4.1. Autoimmune Hematologic Disorders Subsequent to COVID-19 Vaccination

Hematologic AEs subsequent to SARS-CoV-2 vaccine exposition are rare, and can be categorized into three types: autoimmunity exacerbations, AEs secondary to allergic reactions, and inflammatory syndromes triggered by complement activation (reviewed in [17]). Hematologic autoimmune phenomena of new onset, especially those involving platelets, are among the AEs that have been given more attention. Reports are being increasingly made that document secondary immune thrombocytopenia (ITP), vaccine-induced immune thrombotic thrombocytopenia (VITT), immune thrombotic thrombocytopenic purpura (iTTP), autoimmune hemolytic anemia (AIHA), Evans syndrome, aplastic anemia, antiphospholipid syndrome (APS), catastrophic APS (CAPS) or acquired coagulation disorders, subsequent to COVID-19 vaccination. Table 3 shows the documented cases of some of these entities in the European Union, United Kingdom and Canada up to March 2022 [8,9,10]. Their extremely low incidence prevents us from understanding completely whether autoimmune manifestations are merely coincidental or are caused by vaccines’ active components or excipients [18]. What is known about the association between COVID-19 vaccines and autoimmune hematologic disorders is reviewed below.

### 4.2. Risk of Secondary ITP

#### 4.2.1. Historical Perspective

Vaccines activate immune-mediated mechanisms that induce protective immunity but, as well as infections, they may trigger an autoimmune response that in some cases leads to ITP. This is an autoimmune disorder that causes a decrease in platelet counts due to their accelerated destruction and impaired production. A significant high risk of suffering ITP was found after hepatitis A, varicella, and diphtheria-tetanus-acellular pertussis vaccination [19,20,21]. Moreover, several cases of ITP related to poliomyelitis and influenza vaccines (reviewed in [20]), and after Gardasil 9 vaccination for human papillomavirus (HPV) [22], have been published. The trivalent vaccine, containing attenuated strains of measles, rubella, and mumps viruses (MMR), is also associated with ITP, although the risk is smaller than that induced by natural infection with these viruses [23,24,25]. The temporal relationship between vaccination and autoimmunity phenomena seems to be dependent on the specific vaccine.

Vaccines may induce ITP by several mechanisms. The most likely one involves virally induced molecular mimicry, although the participation of others such as those associated with T cell immune-mediated pathways cannot be ruled out. Furthermore, not only the main compound of the product, i.e., mRNA, viral vector, attenuated virus or viral protein, may be involved in activating autoimmune pathways. Other constituents, such as yeast proteins, adjuvants or preservative diluents, may also play a role. Nevertheless, it is important to remark that the risk of ITP after vaccination is smaller than that of those patients exposed to viruses after natural infection [26,27,28,29,30].

Subjects developing ITP after vaccination usually have a favorable outcome. The presentation is acute and mild, and the percentage of remissions is high. It has been shown to be close to 95% in the period of six months after new onset of ITP subsequent to MMR vaccination, with no long-term sequelae, although therapy with corticosteroids or intravenous immunoglobulins (IVIG) may be required [19]. On the other hand, whether or not vaccination can exacerbate thrombocytopenia in patients who already have ITP is less understood, and remains a concern. Importantly, 65 children who had developed ITP after receiving the first dose of the MMR vaccine did not have a recurrence upon receipt of the second one. Nevertheless, it is advisable to delay vaccination until resolution of acute ITP [31,32,33].

#### 4.2.2. COVID-19, COVID-19 Vaccination and ITP

COVID-19 infection is associated with the generation of autoantibodies and the new onset of autoimmune diseases, among which ITP has been reported [34,35,36,37]. Thrombocytopenia, albeit usually mild, has been identified in up to 36% of COVID-19 patients [37]. An important proportion of the infrequent cases of severe thrombocytopenia corresponds to patients who develop ITP upon infection [38].

Although thrombocytopenia secondary to COVID-19 vaccination had not been reported as a frequent AE in the clinical trials that led to the approval of vaccines [12,39], soon afterwards, regular updates of databases highlighted occasional secondary thrombocytopenia appearance in previously healthy recipients when vaccines started being used widely in the general population (Table 3). Several of these cases, which also included fatalities, were reported [40,41,42]. One of the largest series was taking data from up to four different sources: seventy-seven new cases of ITP were reported in March 2021. Thirty-seven and 40 of these subjects had been administered BNT162b2 and mRNA-1273, respectively. The overall incidence was calculated to be ~1 case per million doses. Seventy-seven percent of these subjects developed ITP after receiving the first dose of the vaccine, while 23% developed it after the second one. The median (IQR) platelet count was 3 × 10^9^/L [1,2,3,4,5,6,7,8,9], at a median (IQR) of eight days after vaccination [3,4,5,6,7,8,9,10,11,12,13]. Nevertheless, about 90% of subjects responded to the usual first-line treatments [42]. 

The association between the use of BNT162b2 or mRNA-1273 with the sporadic onset of ITP may be explained by the fact that these are mRNA-based vaccines. mRNA is known to be a potent activator of autoimmunity. mRNA is taken up by cellular RNA receptors, which leads to the upregulation of toll-like receptors (TLR) 7 and 8, and the subsequent activation and maturation of immune cells, as well as the secretion of cytokines and chemokines. The lipid nanoparticle component of mRNA-based COVID-19 vaccines further contributes to immune activation [43,44]. Nevertheless, non-mRNA vaccines might also induce ITP, whose onset could be partly contributed to by residual traces from the manufacturing process, whether adjuvants or preservatives [45,46]. In fact, a new syndrome has been described, so called autoimmune/autoinflammatory syndrome, which is induced by adjuvants (ASIA). It is hypothesized that ITP onset after immunization may be an epiphenomenon of such an entity [47]. In any case, the association of SARS-CoV-2 vaccination with ITP of new onset should not constitute a concern. A study carried out in the USA identified 15 and 13 cases of thrombocytopenia after administration of 18,841,309 and 16,260,102 doses of BNT162b2 and mRNA-1273 vaccines, respectively, which yields a rate of thrombocytopenia of 0.80 per million doses for both of them. Since the annual incidence rate of ITP is 3.3 cases per 100,000 adults, the observed number of thrombocytopenia cases, which includes ITP, among those subjects administered mRNA-based COVID-19 vaccines is not greater than the number of ITP cases expected [48].

Finally, the concern remains that patients with chronic ITP may suffer exacerbations upon SARS-CoV-2 vaccination. Cases have been documented in several reports [33,42,49]. In a case-series that recruited 52 consecutive chronic ITP patients, thrombocytopenia exacerbations were documented in 12% of them, mainly within two to five days post-vaccination and with new bleeding symptoms. ITP exacerbation occurred independently of remission status, concurrent ITP treatment and, importantly, vaccine type [33]. More recently, the analysis of more than 200 previously diagnosed ITP patients that were administered either BNT162b2, mRNA-1273 or ChAdOx1 nCoV-19 vaccine, revealed ITP exacerbations in nearly 14% of cases, bleeding episodes being reported in 2% of patients. Apparently, the type of vaccine did not influence the outcome, although the limited size of the cohort precluded firm conclusions regarding this topic [49].

#### 4.2.3. Management of ITP

Patients with ITP concomitant with COVID-19 infection whose platelet counts fall severely should be administered IVIGs. Platelet transfusion should be given in cases of critical bleeding. On the other hand, there is no evidence suggesting that early treatment with corticosteroids worsens the course of COVID-19 infection. Treatment with dexamethasone in case of severe COVID-19 illness should be given as appropriate. Nevertheless, rituximab and other immunosuppressive agents could impair the ability of the patient to generate their own anti-SARS-CoV2 antibodies. Thrombopoietic agents (TPO-RA) and fostamatinib can also be used in this scenario, and their concomitant administration could be considered. Finally, tromboprophylaxis should be initiated in hospitalized patients with platelet counts above 20–30,000/µL, or with lower counts in the case of a high risk of thromboembolism [50].

**Figure 1:**
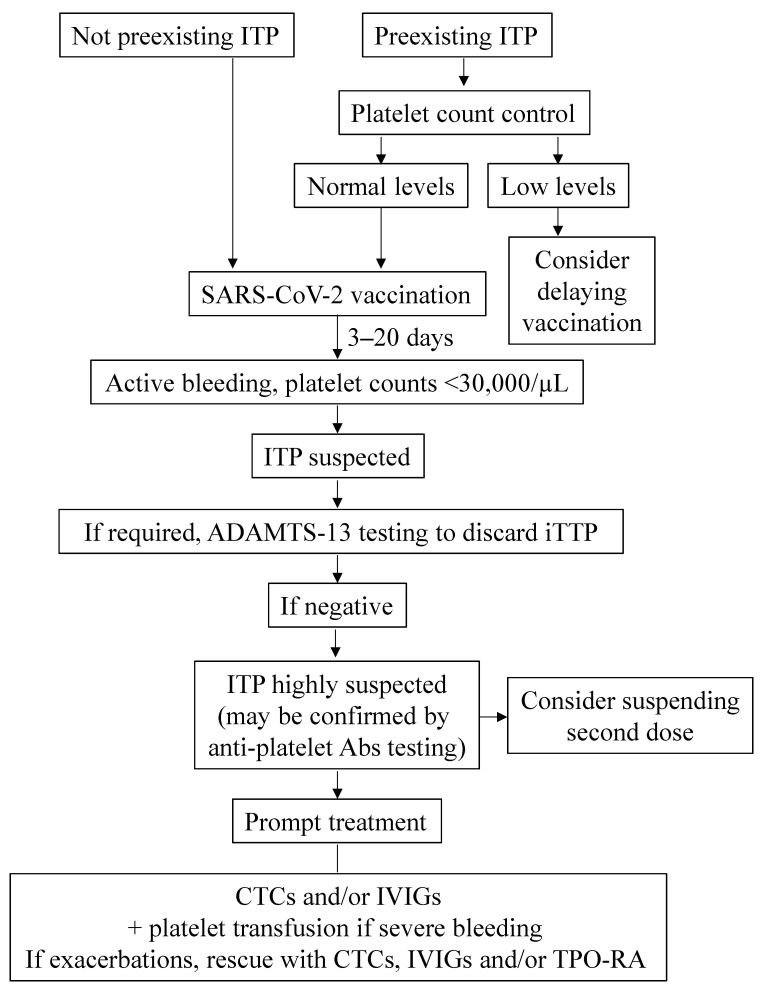
**Management of ITP in the context of COVID-19 vaccination**; Abs, antibodies; CTCs, corticosteroids; iTTP, immune thrombotic thrombocytopenic purpura; IVIGs, intravenous immunoglobulins; TPO-RA, thrombopoetin receptor agonists.

Subjects with ITP subsequent to vaccination respond appropriately to canonical first-line therapy. Figure 1 shows the main steps that may be recommended to follow in the light of current knowledge. In one of the aforementioned studies, 26 out of 28 patients responded to treatment with corticosteroids and/or IVIGs, and/or platelet transfusion. In the same cohort, 19 out of 109 patients with preexisting ITP had exacerbations upon SARS-CoV-2 vaccination. Seven of them were administered rescue therapy with either corticosteroids, TPO-RA, IVIG or a combination of IVIG, steroids, rituximab and cyclosporine, together with the increased dosing of ongoing ITP treatment. All of them responded appropriately, and platelets returned to prevaccination ranges within two to four weeks, with no major bleeding events documented [42].

Key concepts

- Cases of ITP associated with SARS-CoV-2 infection have been reported. Although infrequently, severe thrombocytopenia has been documented among these subjects.
- ITP onset subsequent to vaccination against SARS-CoV-2 has been documented. The overall incidence is extremely low. Exacerbations have been observed in a low proportion of previously diagnosed ITP patients.
- Treatment of secondary ITP or ITP exacerbations subsequent to vaccination is essentially similar to that used in patients with primary ITP. The outcome is usually favorable.

### 4.3. Risk of VITT

#### 4.3.1. Newly Described Autoimmune Disorder

VITT (vaccine-induced immune thrombocytopenia with thrombosis), also known as thrombosis with thrombocytopenia syndrome (TTS) and vaccine-induced prothrombotic immune thrombocytopenia (VIPIT), is a newly described disorder that has been associated with the use of SARS-CoV-2 vaccines. Cases of thrombocytopenia accompanied by unusual thrombotic events have been described to occur 5 to 20 days after vaccination, especially with adenoviral vector-based vaccines. The first report in Europe identified 11 patients in Germany and Austria who had received the ChAdOx1 nCoV-19 vaccine [51]. On the other hand, 12 cases were documented in the USA after the administration of approximately seven million doses [52]. Cases have also been described with mRNA-based BNT162b2 and mRNA-1273, albeit less frequently [53,54]. VITT has not been reported with other adenoviral vectored COVID-19 vaccines such as Gam-COVID-Vac or Ad5-nCoV, although available data are more limited with these compounds. The incidence rate of VITT ranges between one in 125,000 to one in a million vaccinated people [55]. It is important to note that the risk of development of VITT after vaccination against SARS-CoV-2 is lower than the thrombotic risk caused by the disease itself [56].

The vaccine-induced response against SARS-CoV-2 spike protein is not involved in VITT development [57]. In fact, the hallmark of VITT is the presence of high-titer immunoglobulin G (IgG) antibodies directed against the cationic platelet chemokine platelet factor 4 (PF4), which is able to activate platelets. Thrombi are formed in atypical locations, such as the cerebral and splenic vascular beds, and patients often show laboratory signs of disseminated intravascular coagulation (DIC) with severe thrombocytopenia [51,58].

#### 4.3.2. VITT and Heparin-Induced Thrombocytopenia

VITT shows similarities with heparin-induced thrombocytopenia (HIT), both disorders being associated with the presence of autoantibodies against PF4, thrombocytopenia and thrombosis. PF4 is a positively charged protein found in the α-granules of platelets, which is released into plasma when platelets are activated. Although HIT also presents with venous and arterial ischemic events, it differs from VITT in that it is triggered by a recent exposure to heparin, and the autoimmune component is not exactly the same [59]. When PF4 binds to heparin, conformational changes in PF4 occur and a new antigen is exposed, resulting in the development of IgG autoantibodies against the complex formed by heparin and PF4. Once autoantibodies are incorporated into the complex, this crosslinks with FcγRIIa receptors on platelets, resulting in intracellular signaling, platelet activation, and aggregation. In turn, activated platelets degranulate and release more PF4 molecules and procoagulant microparticles into the plasma, thus increasing thrombin generation. The participation of neutrophil extracellular traps, red blood cells, von Willebrand factor, and tissue factor generates a positive feedback loop which results in a hypercoagulable state [60].

Patients with VITT have also high levels of circulating anti-PF4 antibodies that induce variable degrees of platelet activation. However, unlike what happens in HIT, such activation has been shown to be inhibited by low-molecular-weight heparin. It is hypothesized that PF4 would bind to an unidentified polyanion present in adenoviral vaccines. PF4 may also bind to free nucleic acids, since the ability of DNA and RNA to form multimolecular complexes with PF4 has been reported [61]. The formed complexes would be targeted by autoantibodies [60].

#### 4.3.3. Management of VITT

**Figure 2:**
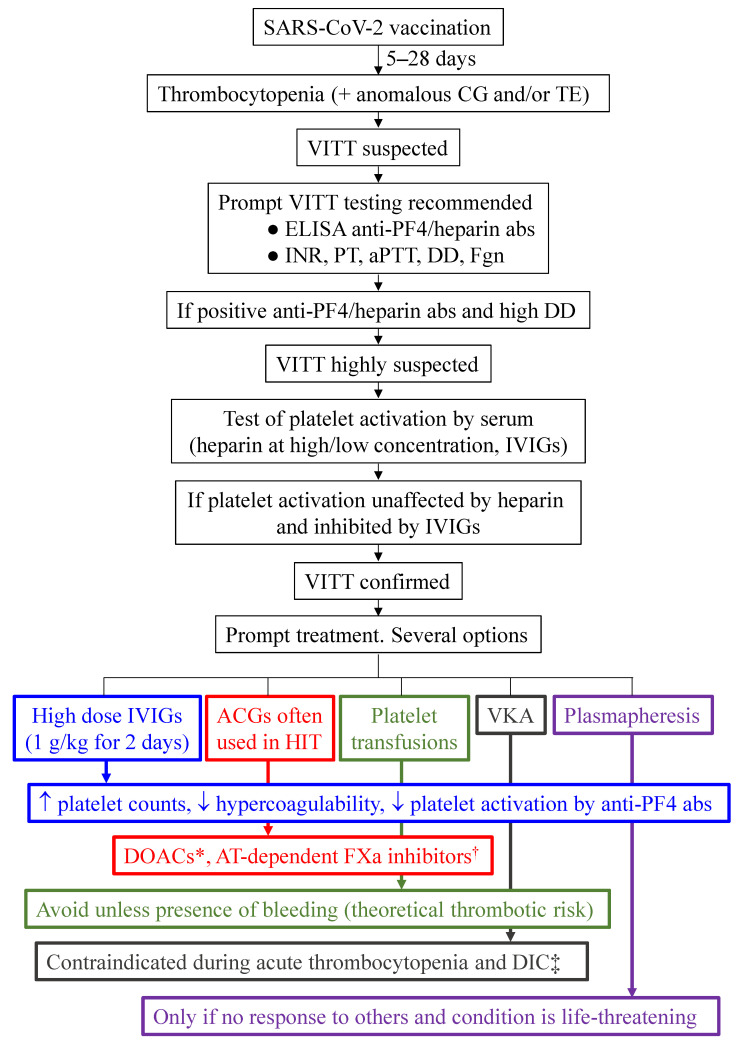
**Management of subjects with suspected VITT after COVID-19 vaccination**; * Direct factor Xa inhibitors (apixaban, rivaroxaban), direct thrombin inhibitors (argatroban, bivalirudin). ^†^ Danaparoid, fondaparinux. ‡ Risk of microthrombosis associated with protein C depletion.

Basically, management should address modulation of the autoimmune phenomenon and non-heparin anticoagulation [62]. Figure 2 summarizes the management of VITT subsequent to SARS-CoV-2 vaccination, from first suspicion to treatment start. Treatment should be promptly initiated in those patients with suspected or confirmed VITT. The International Society on Thrombosis and Haemostasis Scientific Subcommittee (ISTH SSC) on Platelet Immunology has provided recommendations on how to recognize, diagnose, and manage these patients [63]. Early diagnosis and management is important to prevent fatalities, especially when VITT presents with cerebral venous sinus thrombosis (CVST). The documented mortality due to CVST with thrombocytopenia after vaccination with adenoviral vector-based COVID-19 vaccines has experienced a relevant decrease over time. A study carried out with information provided by the EudraVigilance database of the European Medicines Agency involved 270 cases of CVST with thrombocytopenia occurring after adenoviral vector SARS-CoV-2 vaccination. The reported mortality before and after 28 March 2021 was 47% and 22%, respectively, which suggests a positive effect of early recognition and improved treatment on VITT outcomes [64].

The diagnosis procedure should start when the subject presents with thrombocytopenia and altered coagulation, with or without thrombosis, in the four weeks after vaccination. In addition to determining blood cell count, prothrombin time (PT), activated partial thromboplastin time (aPTT), fibrinogen, and D-dimer (DD), the following guidelines should be considered:

- all samples for testing for VITT must be collected prior to administration of any treatment, especially IVIGs and danaparoid.
- assessment of anti-PF4 antibodies should be performed by enzyme-linked immunosorbent assay (ELISA), since rapid tests and chemiluminescence immunoassays can yield false negatives.
- the ability of a patient´s serum to activate platelets must be tested.
- the clinical picture of the individual must be taken into account:
- thrombocytopenia, bleeding, and normal coagulation parameters may indicate ITP. In this case, the bleeding risk has to be considered. High-dose IVIGs without anticoagulation is recommended, as well as testing for autoantibodies against platelet glycoproteins via monoclonal antibody immobilization of platelet antigens (MAIPA), monoclonal antigen capture ELISA (MACE), platelet antibody bead array (PABA), or flow cytometry. Platelet-associated IgGs are not often measured because of the low sensitivity of the usually available methods.
- thrombocytopenia and thrombosis may indicate VITT. An ELISA to detect anti-PF4 antibodies must be performed. If the PF4 binding assay is positive, or not available, the sample’s ability to activate platelets should be determined using serotonin release assay, platelet aggregation, P-selectin exposure, or others. A positive result in the appropriate clinical context strongly supports a diagnosis of VITT.
- thrombocytopenia without bleeding or thrombosis but abnormal coagulation parameters (at least one among PT, APTT, fibrinogen and DD) may indicate early VITT syndrome.

Key concepts

- VITT is a syndrome whose hallmark are anti-PF4 autoantibodies which may develop after COVID-19 vaccination, especially with adenoviral vectored vaccines, at an extremely rare frequency.
- VITT presents with thrombocytopenia and thrombosis at unusual locations, such as cerebral and splenic vascular beds.
- Early diagnosis and management according to ISTH guidelines is crucial to prevent fatalities.

### 4.4. Risk of iTTP

#### 4.4.1. Historical Perspective

iTTP is a thrombotic microangiopathy (TMA) presenting with microangiopathic hemolytic anemia, thrombocytopenia, and organ impairment. The hallmark is a severe deficiency in ADAMTS-13, the von Willebrand factor-cleaving protease, subsequent to the onset of anti-ADAMTS-13 autoantibodies [65]. Before the pandemic era, development of iTTP had been reported in adult subjects within the two weeks after vaccination, albeit at extremely low frequencies: four cases with vaccines against influenza virus, and one case each with vaccines against rabies virus and pneumococo. In all patients, circulating ADAMTS-13 was below 10% of the normal level, and anti-ADAMTS-13 autoantibodies were detected. The underlying mechanisms by which these vaccines may trigger iTTP are presently unknown. In any case, it is important to remark that the outcome of patients was favorable [66,67,68,69,70,71,72]. 

#### 4.4.2. COVID-19, COVID-19 Vaccination and iTTP

Sporadic cases of iTTP concurrent with SARS-CoV-2 infection have been documented in case series [73] and case reports [74,75,76]. Treatments consisted predominantly of plasma exchange and corticosteroids [73,74,75,76], although rituximab and caplacizumab were also used [73,75]. On the other hand, cases of iTTP secondary to vaccination against SARS-CoV-2 have been regularly uploaded to databases (Table 3). Seventeen of these cases have been described ([77,78,79,80,81,82,83,84,85,86,87,88,89], reviewed in [90]). Thirteen of them corresponded to the first acute episode of iTTP [77,78,80,81,82,83,84,85,86,87], and the remaining ones were relapses of preexisting iTTP [79,88,89]. In ten of the first acute episodes and all of the relapsed ones, the BNT162b2 mRNA vaccine had been administered. The remaining three patients received the adenoviral vectored vaccines ChAdOx1 nCoV-19 (two cases [85,86]) and Ad26.CoV2.S (one case [83]). Symptoms appeared more frequently after the first vaccine dose (in 10 out of 13 newly diagnosed patients and three out of four relapsed patients) rather than after the second one, at a median period of 13 days following administration (the earliest and latest cases were at days five and 37, respectively). Confirmatory diagnosis was made in all cases with the assessment of ADAMTS-13 activity, which was always <10%, and the presence of anti-ADAMTS-13 autoantibodies. Most patients presented with hemorrhagic symptoms along with asthenia.

The molecular mechanisms underlying the onset of iTTP after COVID-19 vaccination are unknown, as it happens with those triggering iTTP following administration of other vaccines. The risk seems to be somewhat higher with BNT162b2. A causal association between vaccination and subsequent iTTP is supported by the timing of events rather than the identification of cross-reactive epitopes between antigens in these vaccines and ADAMTS-13. Such timing is consistent with that corresponding to iTTP onset upon administration of other vaccines [66,67,68,69,70,71,72]. Interestingly, in four subjects whose first dose of BNT162b2 induced notably high titers of neutralizing anti-SARS-CoV-2 IgG antibodies, increased titers of the ADAMTS-13 inhibitor accompanied by a marked decrease in ADAMTS-13 activity were observed [78,79,80,81]. Indeed, further studies are needed to unveil the mechanisms underlying the association between COVID-19 vaccination and iTTP.

#### 4.4.3. Management of iTTP

In all cases, plasma exchange and immunosuppressive therapy with corticosteroids, were initiated immediately. Furthermore, caplacizumab was used in eight patients who rapidly recovered platelet counts [77,78,79,89], and rituximab was given in eight cases to boost immunosuppression [80,81,82,84,85,86,87,89]. The clinical outcome was good in most patients. Nevertheless, one of them died two days after being diagnosed with iTTP, probably by a sudden cardiovascular event [77].

**Figure 3:**
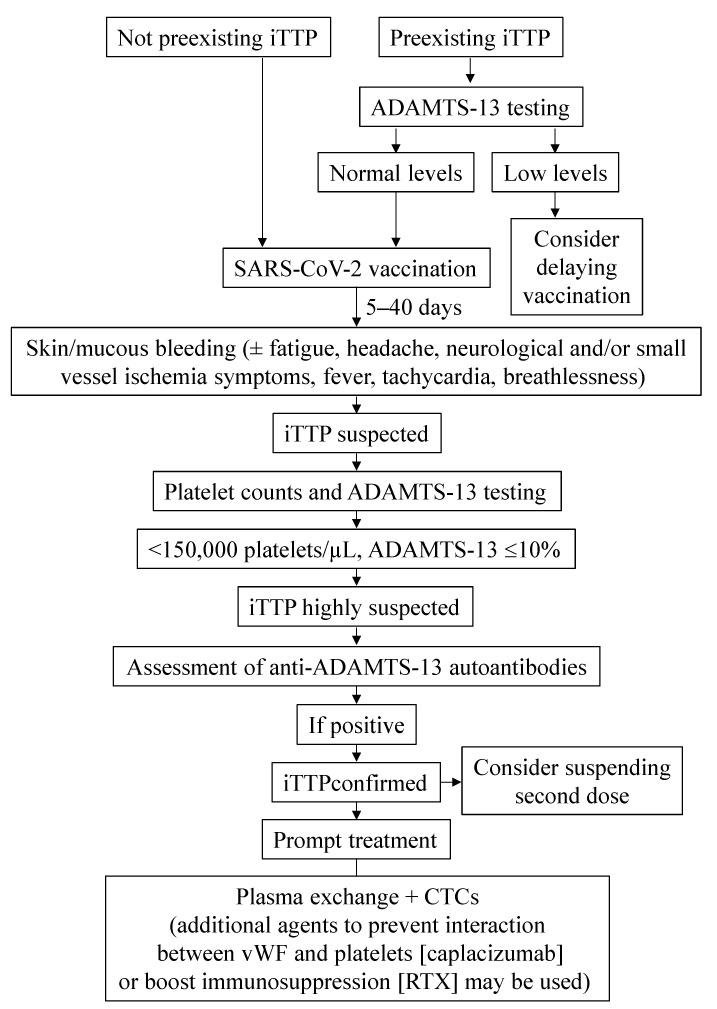
**Management of iTTP in the context of COVID-19 vaccination**; CTCs, corticosteroids; iTTP, immune thrombotic thrombocytopenic purpura; RTX, rituximab.

Finally, the experience provided by these cases, albeit limited, allows for several recommendations regarding the therapeutic approach to follow in scenarios where iTTP is suspected in subjects who have been, or are going to be, administered a COVID-19 vaccine (Figure 3):

- Patients presenting with mucocutaneous bleeding signs and other clinical symptoms such as fatigue, headache and others, after COVID-19 vaccination, should be encouraged to seek immediate medical attention and undergo cell count analyses. Indeed, clinicians should be alert to the risk of developing iTTP.
- In patients who had already suffered an iTTP episode, the ADAMTS-13 circulating activity should be assessed before COVID-19 vaccination in order to address relapse risk.
- It may be advisable not to administer the second dose of the SARS-CoV-2 vaccine to those patients who had an iTTP episode after the first one, although evidence supporting this decision is scarce.

Key concepts

- Extremely infrequent cases of newly diagnosed iTTP subsequent to COVID-19 vaccination, especially with BNT162b2, have been reported. Relapses have also been documented.
- Platelet cell counts and ADAMTS-13 levels have to be assessed if vaccinated patients present with bleeding signs and fatigue and/or other clinical symptoms. A definitive diagnosis is achieved when anti-ADAMTS-13 autoantibodies are detected.
- Management does not differ greatly from that of iTTP in other scenarios, plasma exchange and corticosteroids being the gold-standard treatments. Outcomes are usually favorable.

### 4.5. Risk of AIHA and Evans Syndrome

#### 4.5.1. Historical Perspective

AIHA is an anemic disorder triggered by the generation of autoantibodies against antigens of the red blood cell (RBC) membrane. AIHA is classified into warm antibody hemolytic anemia (WAHA) and cold agglutinin disease (CAD), according to the temperature at which the antibodies interact with RBCs. The origin of AIHA is either idiopathic or linked to immunoproliferative diseases, such as in the case of CAD. The clinical features vary widely, ranging from mild symptoms associated with chronic hemolisis to icteric signs, fever, shock, lumbar pain or dark urine in acute pictures [91]. Evans syndrome is a rare entity, defined by the concomitant or sequential association of AIHA, ITP and, occasionally, immune neutropenia. Evans syndrome is usually linked to other immune disorders, immunodeficiencies, lymphoproliferative syndromes or infections [92]. Cases of AIHA have been occasionally reported in association with oral poliomyelitis, tetanus, diphtheria, pertussis, influenza or pneumococcal vaccines [93,94,95,96,97,98]. The association between vaccination and Evans syndrome is controversial, and only isolated cases have been documented [99,100].

#### 4.5.2. COVID-19, COVID-19 Vaccination and AIHA or Evans Syndrome

Cases of AIHA subsequent to COVID-19 infection have been described. Seven cases of WAHA and CAD were reported within a timeframe compatible with that of the SARS-CoV-2-caused cytokine storm. Interestingly, four out of the seven patients had indolent B lymphoid malignancy [101]. An exacerbation was documented in a patient with a previous history of WAHA, following a recent diagnosis of COVID-19 [102]. A recent review identified up to 50 patients who developed AIHA (either cold, warm or other subtypes) following COVID-19 diagnosis [103]. On the other hand, there are several reports describing cases of newly diagnosed Evans syndrome in the context of COVID-19. Although these are extremely infrequent, the consequences may be severe, since two intracranial bleeding episodes and two venous thromboembolic events were documented (reviewed in [104,105]). 

AIHA has not been considered as an AE associated with SARS-CoV-2 vaccines. However, the VAERS database has already collected more than 200 statements regarding episodes of AIHA subsequent to vaccination (Table 3). Some of the cases are reported in the literature. Four patients developed new onset AIHA following mRNA-based SARS-CoV-2 vaccination. In three of these cases, symptoms started at a median of five days after administration of the first dose (reviewed in [103]). Three new cases of patients who developed symptoms after receiving the first dose of mRNA-1273, the second dose of mRNA-1273, and the third dose of BNT162b2, respectively, have been reported recently [106,107,108]. The mechanism underlying the association is not understood, although the molecular mimicry between ankyrin-1, a red cell membrane protein, and SARS-CoV-2 spike protein might play a role [109]. On the other hand, the number of statements documenting the association between COVID-19 vaccines and Evans syndrome is remarkably low (Table 3). To the best of our knowledge, only one of these cases is described in the literature, corresponding to a female patient who also presented with systemic lupus erythematosus (SLE) after being administered the BNT162b2 vaccine [110].

As far as patients with previous history are concerned, there is very little information available. On the one hand, there is a prospective, observational study addressing post-vaccination exacerbations of ITP in a cohort of patients among whom 10 of them presented with associated AIHA. However, results concerning this subgroup are not provided [42]. On the other hand, in a cohort of 56 patients with a history of AIHA, four elderly patients experienced a clinically significant hemoglobin reduction requiring treatment adjustment after receiving mRNA-based vaccines BNT162b2 or mRNA-1273. The same report described seven patients with autoimmune neutropenia who did not have exacerbations after receiving mRNA-based vaccines [110].

#### 4.5.3. Management of AIHA and Evans Syndrome

**Figure 4:**
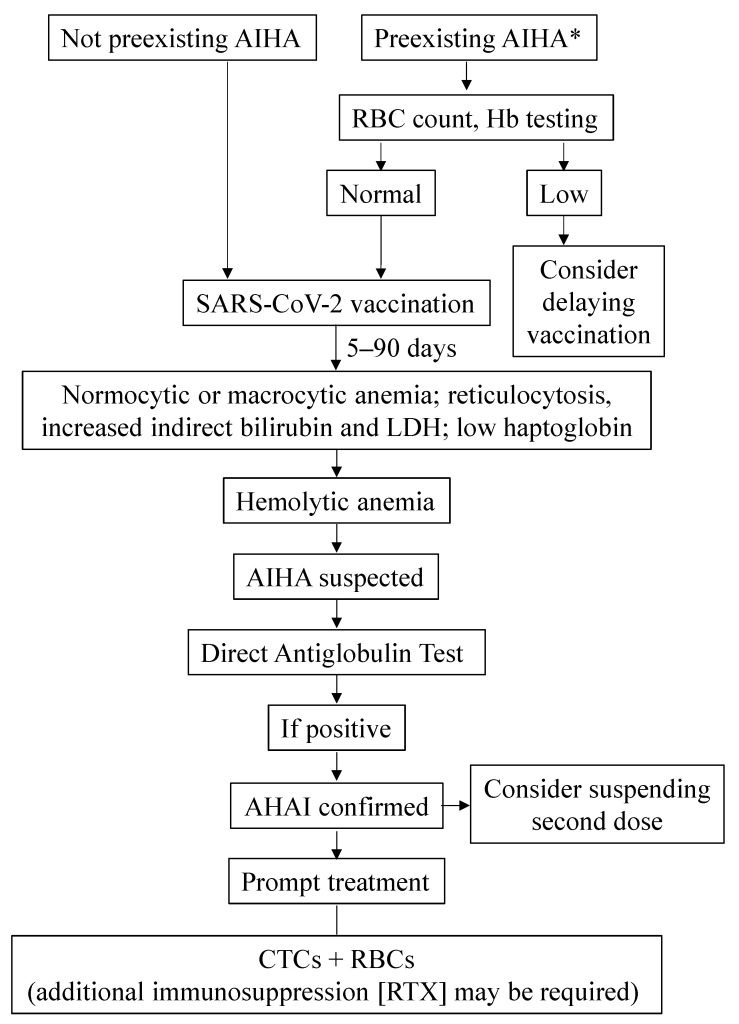
**Management of AIHA in the context of COVID-19 vaccination**; * In case of previous history of Evans syndrome, platelet and neutrophil counts should also be assessed before vaccination. AIHA, autoimmune hemolytic anemia; CTCs, corticosteroids; Hb, hemoglobin; LDH, lactate dehydrogenase; RBCs, red blood cells; RTX, rituximab.

Figure 4 summarizes the actions that may be taken to manage AIHA in the context of SARS-CoV-2 vaccination. Treatment of the reported patients who had AIHA subsequent to COVID-19 infection consisted of corticosteroid administration and RBCs infusion in five and two cases, respectively. The failure of corticosteroid treatment in a patient led to additional rituximab treatment. At the time of last follow-up, this and the rest of the patients were alive and had at least partly recovered from COVID-19 [101]. AIHA exacerbation was initially treated with corticosteroids and folic acid, although additional rituximab and RBCs transfusion had to be administered to prevent hemoglobin decline [102].

AIHA secondary to COVID-19 vaccination was managed with immunosuppressive agents, especially corticosteroids and occasionally rituximab [103,106,107]. A young woman whose case was particularly severe was transfused with 13 units of packed RBCs, and administered corticosteroids, rituximab, mycophenolate mofetil, and immune globulin [108]. Finally, the patient who presented with Evans syndrome and SLE after having received the BNT162b2 vaccine was administered prednisolone to rapidly restore hemoglobin levels and platelet count [110].

Key concepts

- Cases of AIHA subsequent to COVID-19 infection and COVID-19 vaccination, although uncommon, may be severe and life-threatening.
- Corticosteroids and, when required, RBCs transfusion is a usually successful combination to treat patients with AIHA in the context of COVID-19 infection or vaccination. Additional boosts with agents such as rituximab may be occasionally needed.
- Evans syndrome is a rare entity, usually associated with other immune disorders or infection. Information regarding new-onset or exacerbations subsequent to COVID-19 infection or vaccination is scarce. Corticosteroids are considered as the first-line therapy.

#### 4.5.4. Antiphospholipid Syndrome and COVID-19 Vaccines

Patients suffering from antiphospholipid syndrome (APS) have not been included in SARS-CoV-2 vaccination clinical studies, there for little data regarding this population is available [12]. Yu et al., showed that the SARS-CoV-2 spike protein, a key molecule in COVID-19 mRNA vaccine immunogenicity, could be responsible for thrombotic manifestations during COVID-19 disease since it directly activates the alternative complement cascade [111]. Adenoviral vector-based vaccines can bind platelets and induce their destruction in the reticuloendothelial organs. Liposomal mRNA-based vaccines may instead favour the activation of coagulation factors and confer a pro- thrombotic phenotype to endothelial cells and platelets. Furthermore, both formulations may trigger a type I interferon response associated with the generation of antiphospholipid antibodies (aPLs). They may lead to aberrant activation of the immune response with the participation of innate immune cells, cytokines and the complement cascade. NETosis, monocyte recruitment and cytokine release may further support endothelial dysfunction and promote platelet aggregation. These considerations suggest that aPLs may represent a thrombotic risk factor following COVID-19 vaccination [112]. These data could support mRNA vaccine as a trigger for APS and catastrophic antiphospholipid syndrome (CAPS), although several case reports of both conditions after SARS-CoV-2 vaccination have been published [113,114,115,116], other serie do not confirm this hypothesis [117]. In this series of high risk APS patients (triple-positive aPL confirmed 12 weeks apart), out of 161 patients interviewed, 18 (11%) had COVID-19. All of them recovered fully without any progression to severe disease or thromboembolic event. One-hundred-forty-six patients received the first (92%) and 129 (80%) received the second dose of vaccine and fifteen patients (9%) were unvaccinated. The vaccines used by these patients were the mRNA-based vaccine by Pfizer-BioNTech (BNT162b2) in 121 patients, the mRNA-based vaccine Moderna (mRNA-1273) in 20 of them and the adenovirus-vectored vaccine produced by Oxford-AstraZeneca (ChAdOx1 nCoV-19) in the last five. No thromboembolic event was described and no increase in APLs levels was detected. No other relevant adverse event has been communicated. Of the 15 (9%) unvaccinated patients, 13 refused vaccination and two were waiting for the booster dose as they were recently affected by the SARS-CoV-2 infection. Among the 13 patients who did not undergo vaccination, 10 refused vaccinations against SARS-CoV-2 due to fear and vaccine disbelief, one patient with associated SLE followed the treating physician′s recommendation against vaccination, one refused vaccination because of high titre positivity for IgM anti spike protein of SARS-CoV-2 despite having never got the infection, and one refused because of the onset of a serious illness.

From a laboratory point of view, it is common that acute viral infections and some vaccines induce false positivity in serologic assays. By now, there is little information about vaccines against SARS-CoV-2 and serologic assays reactivity. In a serie of 35 subjects, Korentzelos et al., described no change in reactivity of β2-glycoprotein IgA and IgG, cardiolipin IgA and IgG for over the course of samples of patients before vaccination, two weeks after each vaccine dose, and monthly thereafter for up to five months. The used assays were Sure-Vue rapid plasma reagin (RPR) test, (Thermo Fisher Scientific, Waltham, MA, USA) and Macro-Vue RPR test (Becton Dickinson) [118]. These data have been validated by other groups, including no modification in previous positive intensity before and after vaccination in patients suffering from APS [119,120]. Despite this, larger longitudinal studies are needed to determine the incidence and window of false reactivity.

Key concepts:

- Adenoviral vector-based vaccines can bind platelets, activate complement and induce APLs.
- In patients suffering from APS, there has not been reported a worse evolution after COVID-19 vaccination.
- Phospholipid serologic assays seem to not be influenced by SARS-CoV-2 vaccines. Despite this, in case of results that do not fit the clinical picture following SARS-CoV-2, vaccination should be repeated.

## 5. Conclusions

Vaccines against SARS-CoV-2, like other ones, are able to induce antibodies against vaccinated subjects self-antigens that trigger mechanisms leading to hematologic autoimmune disease development. Treatments indicated for secondary ITP, iTTP, AIHA or Evans Syndrome are similar to those used when the vaccine is not the trigger, except for the recommendation to avoid rituximab to warrant the proper immunization against SARS-CoV-2. When a hematologic autoimmune disorder develops following vaccination, a prompt and accurate diagnosis is essential to initiate the appropriate treatment and avoid life-threatening bleeding or thromboembolic complications such as VITT. To go on working on registries and the publication of case series, it is crucial to know the incidence of these AEs, learn about their pathophysiology and plan a suitable vaccine schedule for the affected patients. 

## Figures and Tables

**Table 1:** Characteristics, mechanism of production, storage, efficacy, and adverse events of vaccines against COVID-19.

Compound (Trade Name)	Manufacturer	Mechanism	Doses Needed	Interval	Storage (°C)	Efficacy (%)
BNT162b2 (Comirnaty)	Pfizer/BioNTech	mRNA	2	21 d	−70	95
mRNA-1273 (Spikevax)	Moderna	mRNA	2	28 d	−20	94.5
ChAdOx1 nCoV-19 (Vaxzevria)	AstraZeneca/Oxford	AdV-vectored	2	4–12 wk	2–8	70
Ad26.CoV2.S	Johnson & Johnson	AdV-vectored	1	-	2–8	66.3
Gam-COVID-Vac (Sputnik V)	Gamaleya Research Institute	AdV-vectored	2	21 d	−18	92
Ad5-nCoV (Convidecia)	CanSino	AdV-vectored	1	-	−20	65.7
NVX-CoV2373 (Covovax)	Novavax	Protein subunit	2	21 d	−20	89.3
EpiVacCorona (Aurora-CoV)	Vector Institute	Protein subunit	2	21 d	2–8	n.a.
BBIBP-CorV (Covilo)	Sinopharm (Beijing)	Inactivated virus	2	21–28 d	2–8	79
WIBP-CorV	Sinopharm (Wuhan)	Inactivated virus	2	14–21 d	2–8	72.5
Vero cell (CoronaVac)	Sinovac Biotech	Inactivated virus	2	28 d	2–8	50–83.5
BBV152 (Covaxin)	Bharat Biotech	Inactivated virus	2	28 d	2–8	81

AdV, adenovirus; d, days; n.a., not available; wk, weeks.

**Table 2:** Adverse events associated with the most used vaccines in Western countries.

Vaccine	Number of Administered Doses *	Incidence of SAEs (%) *^,†^	Common AEs ^‡^	SAEs ^§^
**BNT162b2**	770,897,374	0.13 (0.05–0.21)	Local pain (>80%) fever (>10%) headache (>50%) fatigue (>60%) myalgia (>40%) arthralgia (>20%), lymphadenopathy (3%) chills (>30%)	ATE/VTE, ITP, TTS, allergic reactions including anaphylaxis, paroxysmal ventricular arrhythmia, syncope, MIS, myocarditis, pericarditis
**mRNA-1273**	203,315,870	0.18 (0.06–0.29)	Local pain (92%) fever (16%) headache (65%) fatigue (70%) myalgia (61%) arthralgia (46%), lymphadenopathy (20%) chills (45%) nausea/vomiting (23%)	ATE/VTE, ITP, TTS, allergic reactions including anaphylaxis, facial swelling, Bell’s palsy, myocarditis, pericarditis
**ChAdOx1 nCoV-19**	120,953,207	0.42 (0.13–0.68)	Local pain (58%) fever (8%) headache (53%) fatigue (53%) myalgia (44%) arthralgia (27%) chills (32%) nausea/vomiting (22%)	ATE/VTE, TTS, ITP, Guillain-Barré syndrome, CLS, CVST without thrombocytopenia, capillary leak syndrome
**Ad26.CoV2.S**	19,219,676	0.26 (0.23–0.29)	Local pain (49%) fever (9%) headache (39%) fatigue (38%) myalgia (33%) nausea/vomiting (14%)	ATE/VTE, TTS, ITP, Guillain-Barré syndrome, capillary leak syndrome

* Until March 2022 in the European Union, United Kingdom and Canada [8,9,10,11]. ^†^ AEs are expressed as median (IQR). ^‡^ In >16 y.o. subjects in clinical trials [12,13,14,15]. ^§^ Information taken from post-authorization reports [8,9,10,11]. AEs, adverse events; ATE, arterial thromboembolism; CLS, capillary leak syndrome; CVST, cerebral venous sinus thrombosis; ITP, immune thrombocytopenia; MIS, multisystem inflammatory syndrome; SAEs, serious adverse events; TTS, thrombosis with thrombocytopenia syndrome; VTE, venous thromboembolism; y.o., years old.

**Table 3:** Incidence of autoimmune hematologic AEs subsequent to COVID-19 vaccination until March 2022.

Vaccine	Doses, n	ITP, n (inc)	TTP, n (inc)	ES, n (inc)	AIHA, n (inc)
**BNT162b2**	770,897,374	424 (0.55)	89 (0.11)	10 (0.01)	124 (0.16)
**mRNA-1273**	203,315,870	24 (0.12)	38 (0.19)	7 (0.03)	46 (0.23)
**ChAdOx1 nCoV-19**	120,953,207	254 (2.10)	51 (0.42)	5 (0.04)	123 (1.02)
**Ad26.CoV2.S**	19,219,676	13 (0.68)	14 (0.73)	0 (0)	4 (0.21)

The number of autoimmune hematologic AEs corresponding to the European Union, United Kingdom and Canada until March 2022 are shown [8,9,10]. AHAI, autoimmune haemolytic anaemia; ES, Evans syndrome; inc, incidence per million doses; ITP, immune thrombocytopenia; TTP, and thrombotic thrombocytopenic purpura.

## Data Availability

The study did not report any data.

## Supplementary Materials

The following supporting information can be downloaded at: https://www.mdpi.com/article/10.3390/vaccines10060961/s1, Table S1: Categories SARS-CoV-2 vaccines that successfully passed phase III trials belong to.

## References

[1] Fiolet T., Kherabi Y., MacDonald C.J., Ghosn J., Peiffer-Smadja N. (2022). Comparing COVID-19 vaccines for their characteristics, efficacy and effectiveness against SARS-CoV-2 and variants of concern: A narrative review. Clin. Microbiol. Infect..

[2] Ghasemiyeh P., Mohammadi-Samani S., Firouzabadi N., Dehshahri A., Vazin A. (2021). A focused review on technologies, mechanisms, safety, and efficacy of available COVID-19 vaccines. Int. Immunopharmacol..

[3] Palatnik-De-Sousa C.B. (2020). What Would Jenner and Pasteur Have Done About COVID-19 Coronavirus? The Urges of a Vaccinologist. Front. Immunol..

[4] Pushparajah D., Jimenez S., Wong S., Alattas H., Nafissi N., Slavcev R.A. (2021). Advances in gene-based vaccine platforms to address the COVID-19 pandemic. Adv. Drug Deliv. Rev..

[5] Wolff J.A., Malone R.W., Williams P., Chong W., Acsadi G., Jani A., Felgner P.L. (1990). Direct gene transfer into mouse muscle in vivo. Science.

[6] Mendonça S.A., Lorincz R., Boucher P., Curiel D.T. (2021). Adenoviral vector vaccine platforms in the SARS-CoV-2 pandemic. NPJ Vaccines.

[7] Azmi F., Fuaad A.A.H.A., Skwarczynski M., Toth I. (2014). Recent progress in adjuvant discovery for peptide-based subunit vaccines. Hum. Vaccines Immunother..

[8] European Medicines Agency (2017). Guide on the Interpretation of Spontaneous Case Reports of Suspected Adverse Reactions to Medicines.

[9] Medicines & Healthcare Products Regulatory Agency (2021). Coronavirus Vaccine Weekly Summary of Yellow Card Reporting. gov.uk. https://www.gov.uk/government/publications/coronavirus-covid-19-vaccine-adverse-reactions/coronavirus-vaccine-summary-of-yellow-card-reporting.

[10] Reported Side Effects Following COVID-19 Vaccination in Canada. https://health-infobase.canada.ca/covid-19/vaccine-safety/#a4.

[11] The Vaccine Adverse Event Reporting System (VAERS). https://wonder.cdc.gov/controller/datarequest/D8;jsessionid=426D8ABB1739155E11D06093CA0D.

[12] Polack F.P., Thomas S.J., Kitchin N., Absalon J., Gurtman A., Lockhart S., Perez J.L., Marc G.P., Moreira E.D., Zerbini C. (2020). Safety and efficacy of the BNT162b2 mRNA COVID-19 vaccine. N. Engl. J. Med..

[13] Baden L.R., El Sahly H.M., Essink B., Kotloff K., Frey S., Novak R., Diemert M.D., Spector S.A., Rouphael N., Creech B. (2021). Efficacy and Safety of the mRNA-1273SARS-CoV-2 Vaccine. N. Engl. J. Med..

[14] Folegatti P.M., Ewer K.J., Aley P.K., Angus B., Becker S., Belij-Rammerstorfer S., Bellamy D., Bibi S., Bittaye M., Clutterbuck E.A. (2020). Safety and immunogenicity of the ChAdOx1 nCoV-19 vaccine against SARS-CoV-2: A preliminary report of a phase 1/2, single-blind, randomised controlled trial. Lancet.

[15] Sadoff J., Gray G., Vandebosch A., Cárdenas V., Shukarev G., Grinsztejn B., Goepfert P.A., Truyers C., Fennema H., Spiessens B. (2021). Safety and Efficacy of Single-Dose Ad26.COV2.S Vaccine against Covid-19. N. Engl. J. Med..

[16] Logunov D.Y., Dolzhikova I.V., Shcheblyakov D.V., Tukhvatulin A.I., Zubkova O.V., Dzharullaeva A.S., Kovyrshina A.V., Lubenets N.L., Grousova D.M., Gam-COVID-Vac Vaccine Trial Group (2021). Safety and efficacy of an rAd26 and rAd5 vector-based heterologous prime-boost COVID-19 vaccine: An interim analysis of a randomised controlled phase 3 trial in Russia. Lancet.

[17] Al-Ali D., Elshafeey A., Mushannen M., Kawas H., Shafiq A., Mhaimeed N., Mhaimeed O., Mhaimeed N., Zeghlache R., Salameh M. (2021). Cardiovascular and haematological events post COVID-19 vaccination: A systematic review. J. Cell. Mol. Med..

[18] Chen Y., Xu Z., Wang P., Li X.M., Shuai Z.W., Ye D.Q., Pan H.F. (2022). New-onset autoimmune phenomena post-COVID-19 vaccination. Immunology.

[19] O’Leary S.T., Glanz J.M., McClure D.L., Akhtar A., Daley M.F., Nakasato C., Baxter R., Davis R.L., Izurieta H.S., Lieu T.A. (2012). The Risk of Immune Thrombocytopenic Purpura After Vaccination in Children and Adolescents. Pediatrics.

[20] Perricone C., Ceccarelli F., Nesher G., Borella E., Odeh Q., Conti F., Shoenfeld Y., Valesini G. (2014). Immune thrombocytopenic purpura (ITP) associated with vaccinations: A review of reported cases. Immunol. Res..

[21] Schmidt N., Maitland H. (2021). Acute Immune Thrombocytopenia following administration of Shingrix recombinant zoster vaccine. Am. J. Hematol..

[22] Johansen S., Lægreid I.J., Ernstsen S.L., Azrakhsh N.A., Kittang A.O., Lindås R., Gjertsen B.T., Vetti N., Mørtberg T.V., Sørvoll I.H. (2021). Thrombosis and thrombocytopenia after HPV vaccination. J. Thromb. Haemost..

[23] Di Pietrantonj C., Rivetti A., Marchione P., Debalini M.G., Demicheli V. (2020). Vaccines for measles, mumps, rubella, and varicella in children. Cochrane Database Syst. Rev..

[24] Andrews N., Stowe J., Miller E., Svanström H., Johansen K., Bonhoeffer J., Hviid A., VAESCO consortium (2012). A collaborative approach to investigating the risk of thrombocytopenic purpura aMer measles–mumps–rubella vaccination in England and Denmark. Vaccine.

[25] Perez-Vilar S., Weibel D., Sturkenboom M., Black S., Maure C., Castro J.L., Bravo-Alcántara P., Dodd C.N., Romio S.A., de Ridder M. (2017). Enhancing global vaccine pharmacovigilance: Proof-of-concept study on aseptic meningitis and immune thrombocytopenic purpura following measles-mumps containing vaccination. Vaccine.

[26] Catani L., Fagioli M.E., Tazzari P.L., Ricci F., Curti A., Rovito M., Preda P., Chirumbolo G., Amabile M., Lemoli R.M. (2006). Dendritic cells of immune thrombocytopenic purpura (ITP) show increased capacity to present apoptotic platelets to T lymphocytes. Exp. Hematol..

[27] Gu D., Chen Z., Zhao H., Du W., Xue F., Ge J., Sui T., Wu H., Liu B., Lu S. (2010). Th1 (CXCL10) and Th2 (CCL2) chemokine expression in patients with immune thrombocytopenia. Hum. Immunol..

[28] Sakakura M., Wada H., Tawara I., Nobori T., Sugiyama T., Sagawa N., Shiku H. (2007). Reduced Cd4+Cd25+ T cells in patients with idiopathic thrombocytopenic purpura. Thromb. Res..

[29] Yu J., Heck S., Patel V., LeVan J., Yu Y., Bussel J.B., Yazdanbakhsh K. (2008). Defective circulating CD25 regulatory T cells in patients with chronic immune thrombocytopenic purpura. Blood.

[30] Zhang J., Ma D., Zhu X., Qu X., Ji C., Hou M. (2009). Elevated profile of Th17, Th1 and Tc1 cells in patients with immune thrombocytopenic purpura. Haematologica.

[31] Black C., Kaye J.A., Jick H. (2003). MMR vaccine and idiopathic thrombocytopaenic purpura. Br. J. Clin. Pharmacol..

[32] Stowe J., Kafatos G., Andrews N., Miller E. (2007). Idiopathic thrombocytopenic purpura and the second dose of MMR: Figure 1. Arch. Dis. Child..

[33] Kuter J.D. (2021). Exacerbation of immune thrombocytopenia following COVID-19 vaccination. Br. J. Haematol..

[34] Dotan A., Muller S., Kanduc D., David P., Halpert G., Shoenfeld Y. (2021). The SARS-CoV-2 as an instrumental trigger of autoimmunity. Autoimmun. Rev..

[35] Zulfiqar A.-A., Lorenzo-Villalba N., Hassler P., Andrès E. (2020). Immune thrombocytopenic purpura in a patient with COVID-19. N. Engl. J. Med..

[36] Levraut M., Ottavi M., Lechtman S., Mondain V., Jeandel P. (2020). Immune thrombocytopenic purpura after COVID-19 infection. Int. J. Lab. Hematol..

[37] Murt A., Eskazan A.E., Yilmaz U., Ozkan T., Ar M.C. (2020). COVID-19 presenting with immune thrombocytopenia: A case report and review of the literature. J. Med. Virol..

[38] Guan W.J., Ni Z.Y., Hu Y., Liang W.H., Qu C.Q., He J.X., Liu L., Shan H., Lei C.L., Hui D.S.C. (2020). China medical treatment expert group for COVID-19 2020. Clinical Characteristics of coronavirus disease in China. N. Engl. J. Med..

[39] Thomas S.J., Moreira E.D., Kitchin N., Absalon J., Gurtman A., Lockhart S., Perez J.L., Pérez Marc G., Polack F.P., Zerbini C. (2021). Safety and Efficacy of the BNT162b2 mRNA COVID-19 Vaccine through 6 Months. N. Engl. J. Med..

[40] Lee E.J., Cines D.B., Gernsheimer T., Kessler C., Michel M., Tarantino M.D., Semple J.W., Arnold D.M., Godeau B., Lambert M.P. (2021). Thrombocytopenia following Pfizer and Moderna SARS-CoV-2 vaccination. Am. J. Hematol..

[41] David P., Dotan A., Mahroum N., Shoenfeld Y. (2021). Immune Thrombocytopenic Purpura (ITP) Triggered by COVID-19 Infection and Vaccination. Isr. Med. Assoc. J..

[42] Lee E.J., Beltrami-Moreira M., Al-Samkari H., Cuker A., DiRaimo J., Gernsheimer T., Kruse A., Kessler C., Kruse C., Leavitt A.D. (2022). SARS-CoV-2 vaccination and ITP in patients with de novo or preexisting ITP. Blood.

[43] Edwards D.K., Jasny E., Yoon H., Horscroft N., Schanen B., Geter T., Fotin-Mleczek M., Petsch B., Wittman V. (2017). Adjuvant effects of a sequence-engineered mRNA vaccine: Translational profiling demonstrates similar human and murine innate response. J. Transl. Med..

[44] Thi T., Suys E., Lee J., Nguyen D., Park K., Truong N. (2021). Lipid-Based Nanoparticles in the Clinic and Clinical Trials: From Cancer Nanomedicine to COVID-19 Vaccines. Vaccines.

[45] Agmon-Levin N., Paz Z., Israeli E., Shoenfeld Y. (2009). Vaccines and autoimmunity. Nat. Rev. Rheumatol..

[46] Israeli E., Agmon-Levin N., Blank M., Shoenfeld Y. (2009). Adjuvants and autoimmunity. Lupus.

[47] Shoenfeld Y., Agmon-Levin N. (2011). ‘ASIA’-autoimmune/inflammatory syndrome induced by adjuvants. J. Autoimmun..

[48] Welsh K.J., Baumblatt J., Chege W., Goud R., Nair N. (2021). Thrombocytopenia including immune thrombocytopenia after receipt of mRNA COVID-19 vaccines reported to the Vaccine Adverse Event Reporting System (VAERS). Vaccine.

[49] Visser C., Swinkels M., van Werkhoven E.D., Croles F.N.N., Noordzij-Nooteboom H.S., Eefting M., Last-Koopmans S.M., Idink C., Westerweel P.E., Santbergen B. (2022). COVID-19 vaccination in patients with immune thrombocytopenia. Blood Adv..

[50] Bussel J., Cines D., Cooper N., Dunbar C., Michel M., Rodeghiero F. COVID-19 and ITP: Frequently Asked Questions. In COVID-19 Resources of the American Society of Hematology. https://www.hematology.org/covid-19/covid-19-and-itp.

[51] Greinacher A., Thiele T., Warkentin T.E., Weisser K., Kyrle P.A., Eichinger S. (2021). Thrombotic Thrombocytopenia after ChAdOx1 nCov-19 Vaccination. N. Engl. J. Med..

[52] See I., Su J.R., Lale A., Woo E.J., Guh A.Y., Shimabukuro T.T., Streiff M.B., Rao A.K., Wheeler A.P., Beavers S.F. (2021). US Case Reports of Cerebral Venous Sinus Thrombosis With Thrombocytopenia After Ad26.COV2.S Vaccination, March 2 to April 21, 2021. JAMA.

[53] Cines D.B., Bussel J.B. (2021). SARS-CoV-2 Vaccine-Induced Immune Thrombotic Thrombocytopenia. N. Engl. J. Med..

[54] Smadja D.M., Yue Q.-Y., Chocron R., Sanchez O., Louet A.L.-L. (2021). Vaccination against COVID-19: Insight from arterial and venous thrombosis occurrence using data from VigiBase. Eur. Respir. J..

[55] Afshar Z.M., Babazadeh A., Janbakhsh A., Afsharian M., Saleki K., Barary M., Ebrahimpour S. (2022). Vaccine-induced immune thrombotic thrombocytopenia after vaccination against COVID-19: A clinical dilemma for clinicians and patients. Rev. Med. Virol..

[56] Warkentin T.E., Cuker A. (2022). COVID-19: Vaccine-Induced Immune Thrombotic Thrombocytopenia (VITT), UpToDate. https://www.uptodate.com/contents/covid-19-vaccine-induced-immune-thrombotic-thrombocytopenia-vitt.

[57] Greinacher A., Selleng K., Mayerle J., Palankar R., Wesche J., Reiche S., Aebischer A., Warkentin T.E., Muenchhoff M., Immune-Response in COVID-19 Vaccination Study Group (2021). Anti-platelet factor 4 antibodies causing VITT do not cross-react with SARS-CoV-2 spike protein. Blood.

[58] Schultz N.H., Sørvoll I.H., Michelsen A.E., Munthe L.A., Lund-Johansen F., Ahlen M.T., Wiedmann M., Aamodt A.-H., Skattør T.H., Tjønnfjord G.E. (2021). Thrombosis and Thrombocytopenia after ChAdOx1 nCoV-19 Vaccination. N. Engl. J. Med..

[59] Greinacher A., Selleng K., Warkentin T.E. (2017). Autoimmune heparin-induced thrombocytopenia. J. Thromb. Haemost..

[60] Rizk J.G., Gupta A., Sardar P., Henry B.M., Lewin J.C., Lippi G., Lavie C.J. (2021). Clinical Characteristics and Pharmacological Management of COVID-19 Vaccine-Induced Immune Thrombotic Thrombocytopenia With Cerebral Venous Sinus Thrombosis: A Review. JAMA Cardiol..

[61] Jaax M.E., Krauel K., Marschall T., Brandt S., Gansler J., Fürll B., Appel B., Fischer S., Block S., Helm C.A. (2013). Complex formation with nucleic acids and aptamers alters the antigenic properties of platelet factor 4. Blood.

[62] Klok F.A., Pai M., Huisman M.V., Makris M. (2022). Vaccine-induced immune thrombotic thrombocytopenia. Lancet Haematol..

[63] Nazy I., Sachs U.J., Arnold D.M., McKenzie S.E., Choi P., Althaus K., Ahlen M.T., Sharma R., Grace R.F., Bakchoul T. (2021). Recommendations for the clinical and laboratory diagnosis of VITT against COVID-19: Communication from the ISTH SSC Subcommittee on Platelet Immunology. J. Thromb. Haemost..

[64] Van de Munckhof A., Krzywicka K., de Sousa D.A., van Kammen M.S., Heldner M.R., Jood K., Lindgren K., Tatlisumak T., Putaala J., Hovinga J.A.K. (2022). Declining mortality of cerebral venous sinus thrombosis with thrombocytopenia after SARS-CoV-2 vaccination. Eur. J. Neurol..

[65] Sadler J.E. (2015). What’s new in the diagnosis and pathophysiology of thrombotic thrombocytopenic purpura. Hematol. Am. Soc. Hematol. Educ. Program.

[66] Yavaşoğlu I. (2020). Vaccination and Thrombotic Thrombocytopenic Purpura. Turk. J. Hematol..

[67] Kadikoylu G., Yavasoglu I., Bolaman Z. (2014). Rabies vaccine-associated thrombotic thrombocytopenic purpura. Transfus. Med..

[68] Kojima Y., Ohashi H., Nakamura T., Nakamura H., Yamamoto H., Miyata Y., Iida H., Nagai H. (2014). Acute thrombotic thrombocytopenic purpura after pneumococcal vaccination. Blood Coagul. Fibrinolysis.

[69] Ramakrishnan N., Parker L.P. (1998). Thrombotic thrombocytopenic purpura following influenza vaccination—A brief case report. Connect. Med..

[70] Dias P.J., Gopal S. (2009). Refractory thrombotic thrombocytopenic purpura following influenza vaccination. Anaesthesia.

[71] Hermann R., Pfeil A., Busch M., Kettner C., Kretzschmar D., Hansch A., La Rosée P., Wolf G. (2010). Very severe thrombotic thrombocytopenic purpura (TTP) after H1N1 vaccination. Med. Klin..

[72] Brown R.C., Blecher T.E., French E.A., Toghill P.J. (1973). Thrombotic thrombocytopenic purpura after influenza vaccination. BMJ.

[73] Tehrani H.A., Darnahal M., Vaezi M., Haghighi S. (2021). COVID-19 associated thrombotic thrombocytopenic purpura (TTP); A case series and mini-review. Int. Immunopharmacol..

[74] Hindilerden F., Yonal-Hindilerden I., Akar E., Kart-Yasar K. (2020). Covid-19 associated autoimmune thrombotic thrombocytopenic purpura: Report of a case. Thromb. Res..

[75] Capecchi M., Mocellin C., Abbruzzese C., Mancini I., Prati D., Peyvandi F. (2020). Dramatic presentation of acquired thombotic thrombocytopenic purpura associated with COVID-19. Haematologica.

[76] Albiol N., Awol R., Martino R. (2020). Autoimmune thrombotic thrombocytopenic purpura (TTP) associated with COVID-19. Ann. Hematol..

[77] Giuffrida G., Condorelli A., Di Giorgio M.A., Markovic U., Sciortino R., Nicolosi D., Di Raimondo F. (2022). Immune-mediated thrombotic thrombocytopenic purpura following administration of Pfizer-BioNTech COVID-19 vaccine. Haematologica.

[78] De Bruijn S., Maes M.B., De Waele L., Vanhoorelbeke K., Gadisseur A. (2021). First report of a de novo iTTP episode associated with an mRNA-based anti-COVID-19 vaccination. J. Thromb. Haemost..

[79] Maayan H., Kirgner I., Gutwein O., Herzog-Tzarfati K., Rahimi-Levene N., Koren-Michowitz M., Blickstein D. (2021). Acquired thrombotic thrombocytopenic purpura: A rare disease associated with BNT162b2 vaccine. J. Thromb. Haemost..

[80] Ruhe J., Schnetzke U., Kentouche K., Prims F., Baier M., Herfurth K., Schlosser M., Busch M., Hochhaus A., Wolf G. (2021). Acquired thrombotic thrombocytopenic purpura after first vaccination dose of BNT162b2 mRNA COVID-19 vaccine. Ann. Hematol..

[81] Yoshida K., Sakaki A., Matsuyama Y., Mushino T., Matsumoto M., Sonoki T., Tamura S. (2022). Acquired Thrombotic Thrombocytopenic Purpura Following BNT162b2 mRNA Coronavirus Disease Vaccination in a Japanese Patient. Intern. Med..

[82] Alislambouli M., Victoria A.V., Matta J., Yin F. (2021). Acquired thrombotic thrombocytopenic purpura following Pfizer COVID-19 vaccination. eJHaem.

[83] Yocum A., Simon E.L. (2021). Thrombotic Thrombocytopenic Purpura after Ad26.COV2-S Vaccination. Am. J. Emerg. Med..

[84] Waqar S.H.B., Khan A.A., Memon S. (2021). Thrombotic thrombocytopenic purpura: A new menace after COVID bnt162b2 vaccine. Int. J. Hematol..

[85] Al-Ahmad M., Al-Rasheed M., Shalaby N.A.B. (2021). Acquired thrombotic thrombocytopenic purpura with possible association with AstraZeneca-Oxford COVID-19 vaccine. EJHaem.

[86] Lee H.P., Selvaratnam V., Rajasuriar J.S. (2021). Thrombotic thrombocytopenic purpura after ChAdOx1 nCoV-19 vaccine. BMJ Case Rep..

[87] Kirpalani A., Garabon J., Amos K., Patel S., Sharma A.P., Ganesan S.L., Barton M., Cacciotti C., Leppington S., Bakovic L. (2021). Thrombotic thrombocytopenic purpura temporally associated with BNT162b2 vaccination in an adolescent successfully treated with caplacizumab. Br. J. Haematol..

[88] Sissa C., Al-Khaffaf A., Frattini F., Gaiardoni R., Mimiola E., Montorsi P., Melara B., Amato M., Peyvandi F., Franchini M. (2021). Relapse of thrombotic thrombocytopenic purpura after COVID-19 vaccine. Transfus. Apher. Sci..

[89] Deucher W., Sukumar S., Cataland S.R. (2022). Clinical relapse of immune-mediated thrombotic thrombocytopenic purpura following COVID-19 vaccination. Res. Pr. Thromb. Haemost..

[90] Saluja P., Gautam N., Yadala S., Venkata A.N. (2022). Thrombotic thrombocytopenic purpura (TTP) after COVID-19 vaccination: A systematic review of reported cases. Thromb. Res..

[91] Barcellini W., Fattizzo B. (2020). The Changing Landscape of Autoimmune Hemolytic Anemia. Front. Immunol..

[92] Audia S., Grienay N., Mounier M., Michel M., Bonnotte B. (2020). Evans’ Syndrome: From Diagnosis to Treatment. J. Clin. Med..

[93] Seltsam A., Shukry-Schulz S., Salama A. (2000). Vaccination-associated immune hemolytic anemia in two children. Transfusion.

[94] Downes K.A., Domen R.E., McCarron K.F., Bringelsen K.A. (2001). Acute Autoimmune Hemolytic Anemia Following DTP Vaccination: Report of a Fatal Case and Review of the Literature. Clin. Pediatr..

[95] Johnson S.T., McFarland J.G., Kelly K.J., Casper J.T., Gottschall J.L. (2002). Transfusion support with RBCs from an Mk homozygote in a case of autoimmune hemolytic anemia following diphtheria-pertussis-tetanus vaccination. Transfusion.

[96] Montagnani S., Tuccori M., Lombardo G., Testi A., Mantarro S., Ruggiero E., Blandizzi C. (2010). Autoimmune Hemolytic Anemia Following MF59-Adjuvanted Influenza Vaccine Administration: A Report of Two Cases. Ann. Pharmacother..

[97] Gani I., Hinnant G., Kapoor R., Savage N. (2019). Autoimmune Hemolytic Anemia in a Renal Transplant Patient Following Seasonal Influenza Vaccination. Case Rep. Hematol..

[98] Bowen L., LePage N., Lewandowska M., Waxman D.A. (2019). Anti-Pr antibody induced cold autoimmune hemolytic anemia following pneumococcal vaccination. Clin. Case Rep..

[99] Shlamovitz G.Z., Johar S. (2013). A Case of Evans’ Syndrome Following Influenza Vaccine. J. Emerg. Med..

[100] Hsieh Y.-L., Lin L.-H. (2010). Thrombocytopenic Purpura Following Vaccination in Early Childhood: Experience of a Medical Center in the Past 2 Decades. J. Chin. Med Assoc..

[101] Lazarian G., Quinquenel A., Bellal M., Siavellis J., Jacquy C., Re D., Merabet F., Mekinian A., Braun T., Damaj G. (2020). Autoimmune haemolytic anaemia associated with COVID-19 infection. Br. J. Haematol..

[102] Finkenthal T.A., Aldaher Z., Ahmed S., DiValentin L. (2021). Autoimmune Hemolytic Anemia Exacerbation Associated With COVID-19 Infection and Markedly Elevated Inflammatory Markers. Cureus.

[103] Jacobs J.W., Booth G.S. (2021). COVID-19 and Immune-Mediated RBC Destruction. Am. J. Clin. Pathol..

[104] Taherifard E., Taherifard E., Movahed H., Mousavi M.R. (2021). Hematologic autoimmune disorders in the course of COVID-19: A systematic review of reported cases. Hematology.

[105] Turgutkaya A., Bolaman A.Z., Yavaşoğlu İ. (2021). COVID-19-associated Evans syndrome: A case report and review of the literature. Transfus. Apher. Sci..

[106] Jaydev F., Kumar V., Khatri J., Shahani S., Beganovic S. (2022). A Case of Autoimmune Hemolytic Anemia after the First Dose of COVID-19 mRNA-1273 Vaccine with Undetected Pernicious Anemia. Case Rep. Hematol..

[107] Fatima Z., Reece B.R.A., Moore J.S., Means R.T. (2022). Autoimmune Hemolytic Anemia After mRNA COVID Vaccine. J. Investig. Med. High Impact Case Rep..

[108] De Bruyne S., Van Landeghem S., Schauwvlieghe A., Noens L. (2022). Life-threatening autoimmune hemolytic anemia following mRNA COVID-19 vaccination: Don’t be too prudent with the red gold. Clin. Chem. Lab. Med..

[109] Angileri F., Légaré S., Gammazza A.M., de Macario E.C., Macario A.J.L., Cappello F. (2020). Is molecular mimicry the culprit in the autoimmune haemolytic anaemia affecting patients with COVID-19?. Br. J. Haematol..

[110] Hidaka D., Ogasawara R., Sugimura S., Fujii F., Kojima K., Nagai J., Ebata K., Okada K., Kobayashi N., Ogasawara M. (2021). New-onset Evans syndrome associated with systemic lupus erythematosus after BNT162b2 mRNA COVID-19 vaccination. Int. J. Hematol..

[111] Fattizzo B., Giannotta J.A., Cecchi N., Barcellini W. (2021). SARS-CoV-2 vaccination in patients with autoimmune cytopenias: The experience of a reference center. Am. J. Hematol..

[112] Yu J., Yuan X., Chen H., Chaturvedi S., Braunstein E.M., Brodsky R.A. (2020). Direct activation of the alternative complement pathway by SARS-CoV-2 spike proteins is blocked by factor D inhibition. Blood.

[113] Talotta R., Robertson E.S. (2021). Antiphospholipid antibodies and risk of post-COVID-19 vaccination thrombophilia: The straw that breaks the camel’s back?. Cytokine Growth Factor Rev..

[114] Moreno-Torres V., Gutiérrez Á., Valdenebro M., Ortega A., Cítores M.J., Montero E. (2022). Catastrophic antiphospholipid syndrome triggered by mRNA COVID-19 vaccine. Clin. Exp. Rheumatol..

[115] Molina Rios S., Rojas Martinez R., Estévez Ramirez G.M., Medina Y.F. (2022). Systemic Lupus Erythematosus and Antiphospholipid Syndrome After COVID-19 Vaccination. A Case Report. Mod. Rheumatol. Case Rep..

[116] Jinno S., Naka I., Nakazawa T. (2021). Catastrophic antiphospholipid syndrome complicated with essential thrombocythaemia after COVID-19 vaccination: In search of the underlying mechanism. Rheumatol. Adv. Pract..

[117] Pengo V., Del Ross T., Tonello M., Andreoli L., Tincani A., Gresele P., Silvestri E., Simioni P., Campello E., Hoxha A. (2022). Impact of COVID-19 and COVID-19 vaccination on high-risk patients with Antiphospholipid Syndrome: A nationwide survey. Rheumatology.

[118] Korentzelos D., Baloda V., Jung Y., Wheeler B., Shurin M.R., Wheeler S.E. (2022). COVID-19 mRNA Vaccines May Cause False Reactivity in Some Serologic Laboratory Tests, Including Rapid Plasma Reagin Tests. Am. J. Clin. Pathol..

[119] Signorelli F., Balbi G.G.M., Aikawa N.E., Silva C.A., Kupa L.V.K., Medeiros-Ribeiro A.C., Yuki E.F., Pasoto S.G., Saad C.G., Borba E.F. (2022). Immunogenicity, safety, and antiphospholipid antibodies after SARS-CoV-2 vaccine in patients with primary antiphospholipid syndrome. Lupus.

[120] Thurm C., Reinhold A., Borucki K., Kahlfuss S., Feist E., Schreiber J., Reinhold D., Schraven B. (2022). Homologous and Heterologous Anti-COVID-19 Vaccination Does Not Induce New-Onset Formation of Autoantibodies Typically Accompanying Lupus Erythematodes, Rheumatoid Arthritis, Celiac Disease and Antiphospholipid Syndrome. Vaccines.

