# Supplementary material for: COVID-19 Vaccines and Autoimmune Hematologic Disorders
Source: Vaccines (Basel). 2022 Jun 16;10(6):961. doi: 10.3390/vaccines10060961 (PMC9231220; doi:10.3390/vaccines10060961)
Supplement: Supplementary file 1 [file vaccines-10-00961-s001.zip › vaccines-1759350-supplementary.pdf]

Supplementary Table S1. Categories SARS-CoV-2 vaccines that successfully passed phase III trials belong to

Type of vaccine	Product name
**Inactivated virus**	NVX-COV2373, CoronaVac, BBIBP-CorV, Wuhan Sinopharm inactivated vaccine, Covaxin, QazVac, KoviVac, COVIran Barekat
**Protein encoding mRNA**	BNT162b2*, mRNA-1273*, CVnCoV
**Protein encoding plasmid DNA vector**	INO-4800, AG0301-COVID19, ZyCoV-D, GX-19, ZyCoV-D, bacTLR-Spike, CORVax12
**Protein encoding viral vector**	AZD1222, Sputnik V, Sputnik V Light, Ad5-nCoV (Convidecia), Ad26.COV2.S
**Protein/polysaccharides**	EpiVacCorona, ZF2001, Abdala

*First authorized SARS-CoV-2 mRNA-based vaccines.
